# 2023 List of Reviewers

**DOI:** 10.1117/1.NPh.11.1.010102

**Published:** 2024-01-12

## Abstract

Thanks to reviewers who served Neurophotonics in 2023.

*Neurophotonics* would like to sincerely thank the following individuals who served as reviewers in 2023. The success of our publication hinges on the voluntary contributions of time and energy put forth by these professionals.

Androu Abdalmalak
Lamiae Abdeladim
Nicolo Accanto
Daniel Aharoni
Anna Letizia Allegra Mascaro
Lyubov Amitonova
Wesley Baker
Bradley Baker
Matilde Balbi
Zeinab Barati
Adam Bauer
Jean-Claude Béïque
Ken Berglund
Renzhe Bi
Borja Blanco
Anna Blasi
Catherine Bouchard
Sabrina Brigadoi
Joshua Brumberg
Chiara Bulgarelli
Robert Campbell
Stefan Carp
Hsi Chang
Siyu Chen
Minghai Chen
Xiaojun Cheng
Nicole Chernavsky
Shih-Hsuan Chia
Regine Choe
Vanessa Coelho-Santos
Liam Collins-Jones
Daniel Côté
Ivan Coto Hernandez
Joao Couto
Marco Dal Maschio
Hod Dana
Helga De Oliveira Miguel
Edouard Delaire
Eugene Dempsey
Minghao Dong
Zhe Dong
Tanja Dragojevic
Patrick Drew
Yang Du
Yang Du
Jeff Dunn
Marti Duocastella
Adam Eggebrecht
Christian Eggeling
Aykut Eken
Marc Ekker
Tae Joong Eom
Deano Farinella
Tom Flossmann
Amanda Foust
Ron Frostig
Xiangham Gao
Yuanyuan Gao
Jessica Gemignani
Judit Gervain
Ariel Gilad
Grant Gordon
Gorm Greisen
Dirk Grosenick
Yong Gu
Edgar Guevara
Changliang Guo
Eugenio Gutierrez
Kurt Haas
Allison Hamilos
Lutfu Hanoglu
Fritjof Helmchen
Takatoshi Hikida
Brian Hill
Riichiro Hira
Roger Ho Chun Man
Ute Hochgeschwender
Jie Huang
Sagi Jaffe-Dax
Na Ji
Hongbo Jia
Woonggyu Jung
Rini Kaplan
Deepa Kasaragod
Hiroshi Kawaguchi
Alfred Kaye
Ya Ke
Anastasia Kerr-German
Mehdi Khoshboresh-Masouleh
Kıvılcım Kılıç
Tiffany Ko
Lisa Kobayashi
Sri-Rajasekhar Kothapalli
Minoru Koyama
Alexxai Kravitz
Takahiro Kuchimaru
Bernd Kuhn
Thomas Kuner
Alex Kwan
Baptiste Lacoste
Vittorino Lanzio
Nicole Lazar
Jérôme Lecoq
Seung Yup Lee
Lei Li
Gangjun Liu
Rui Liu
Pablo Loza-Alvarez
Harold MacGillavry
Nicholas MacKinnon
David Margolis
Kazuto Masamoto
Lev Matveev
Guanghan Meng
Frederic Meunier
Daniel Milej
David Miller
Miguel Mireles Nuñez
Mohammad Moeini
Aseema Mohanti
Samuel Montero-Hernandez
Jutta Mueller
Jeremy Murphy
Timothy Murphy
Margaret Naeser
Takeharu Nagai
Noman Naseer
Thien Nguyen
Muhammad Khalid Khan Niazi
Ilkka Nissila
Haijing Niu
Sergio Novi Junior
Yuki Ogawa
Justin O’Hare
Philip OHerron
Felipe Orihuela-Espina
Dan Oron
Francesca Palombo
Vimal Prabhu Pandiyan
Eirini Papagiakoumou
Jongchan Park
Jones Parker
Ke Peng
Paola Pinti
Filippo Pisano
Martin Ploschner
Christophe Proulx
Vidhya Rangaraju
Rebecca Re
Patrick Reeson
Tobias Rose
Ed Ruthazer
Takashi Saitou
Sava Sakadzic
Masayuki Sakamoto
Shuzo Sakata
Hendrik Santosa
Markus Sauer
Travis Sawyer
Giuliano Scarcelli
Felix Scholkmann
Eric Schreiter
Derek Sederman
Lucas Sjulson
Sruthi Srinivasan
Keith St. Lawrence
Jillian Stobart
Aleh Sudakou
Ulas Sunar
Jianbo Tang
Jiannis Taxidis
Lei Tian
Maria Torres-Mapa
Jason Trobaugh
Hidekazu Tsutsui
Daisuke Tsuzuki
Sergey Turtaev
Tara Urner
Matthieu Vanni
Ernesto Vidal Rosas
Alexander Von Luehmann
Fabrice Wallois
Hui Wang
Quan Wang
Martin Wolf
Jingyi Wu
Melissa Wu
Lei Xi
Sheng Xiao
Yijing Xie
Yi Xue
Weijian Yang
Mohammad Yaseen
Andy Yeh
Eric Yttri
Guoqiang Yu
Mark Zarella
Frederick Zeiler
Hongting Zhao
Hubin Zhao
Yongxin Zhao
Haining Zhong
Hamoon Zohdi
Weijian Zong

